# Heptamethine Cyanine-Loaded Nanomaterials for Cancer Immuno-Photothermal/Photodynamic Therapy: A Review

**DOI:** 10.3390/pharmaceutics14051015

**Published:** 2022-05-08

**Authors:** Cátia G. Alves, Rita Lima-Sousa, Bruna L. Melo, André F. Moreira, Ilídio J. Correia, Duarte de Melo-Diogo

**Affiliations:** 1 CICS-UBI—Centro de Investigação em Ciências da Saúde, Universidade da Beira Interior, 6200-506 Covilha, Portugal; catia.g.alves@ubi.pt (C.G.A.); anaritalimasousa1994@gmail.com (R.L.-S.); bdanielalmelo@gmail.com (B.L.M.); afmoreira@fcsaude.ubi.pt (A.F.M.); 2 CIEPQPF–Departamento de Engenharia Química, Universidade de Coimbra, Rua Sílvio Lima, 3030-790 Coimbra, Portugal

**Keywords:** cancer, heptamethine cyanines, immunotherapy, nanoparticles, phototherapies

## Abstract

The development of strategies capable of eliminating metastasized cancer cells and preventing tumor recurrence is an exciting and extremely important area of research. In this regard, therapeutic approaches that explore the synergies between nanomaterial-mediated phototherapies and immunostimulants/immune checkpoint inhibitors have been yielding remarkable results in pre-clinical cancer models. These nanomaterials can accumulate in tumors and trigger, after irradiation of the primary tumor with near infrared light, a localized temperature increase and/or reactive oxygen species. These effects caused damage in cancer cells at the primary site and can also (i) relieve tumor hypoxia, (ii) release tumor-associated antigens and danger-associated molecular patterns, and (iii) induced a pro-inflammatory response. Such events will then synergize with the activity of immunostimulants and immune checkpoint inhibitors, paving the way for strong T cell responses against metastasized cancer cells and the creation of immune memory. Among the different nanomaterials aimed for cancer immuno-phototherapy, those incorporating near infrared-absorbing heptamethine cyanines (Indocyanine Green, IR775, IR780, IR797, IR820) have been showing promising results due to their multifunctionality, safety, and straightforward formulation. In this review, combined approaches based on phototherapies mediated by heptamethine cyanine-loaded nanomaterials and immunostimulants/immune checkpoint inhibitor actions are analyzed, focusing on their ability to modulate the action of the different immune system cells, eliminate metastasized cancer cells, and prevent tumor recurrence.

## 1. Introduction

Cancer survival is, in many cases, a mirage due to metastization processes and tumor relapse [1]. This harsh reality is inherently correlated with the inadequacy of classical treatments (e.g., surgery, chemotherapy, radiotherapy) to completely eliminate metastasized cancer cells and to trigger the creation of immune memory [1,2]. To tackle these problems, researchers and clinicians have focused on developing strategies that can re-engage the immune system in the fight against local and metastasized cancer cells [1,3]. In this regard, nanomaterial-mediated immuno-phototherapy has been yielding remarkable results in preclinical models [4,5,6].

This promising therapeutic modality explores the nanomaterials’ physicochemical features for enabling tumor uptake, as well as their optical properties, which strongly influence the phototherapeutic outcome [7,8,9]. Upon irradiation of the primary tumor with light, the primary tumor-homed nanomaterials can absorb its energy, producing heat (photothermal therapy (PTT)) and/or reactive oxygen species (ROS; photodynamic therapy (PDT)) [10,11]. In brief, the photoresponsive agent absorbs light energy and is transformed into an excited state. Subsequently, part of this energy can then be released through fluorescence and heat emissions [12,13]. The excited photoresponsive agent can also go to a more stable triplet state, which can then react with oxygen, forming singlet oxygen, or react with endogenous molecules to form radicals that interact with oxygen, yielding other ROS (superoxide anion, hydroxyl radical, hydrogen peroxide) [12,13,14,15].

In general, photothermal heating to temperatures around 41–45 °C can induced reversible damage to cells (e.g., affect DNA repair mechanisms or metabolic pathways) or sensitize them to the action of other agents (e.g., chemotherapeutic drugs) [16]. In turn, hyperthermia of 50 °C (or above) causes irreversible damage in cells in the primary tumor (e.g., mitochondrial/enzymatic dysfunctions, protein denaturation, membrane destruction), culminating in cell death by necrosis—reviewed in detail in [16,17]. Furthermore, the photogenerated ROS are highly reactive and short-lived, inducing oxidative stress and damage to the nearby structures, leading to cell death by apoptosis (most common), necrosis, and autophagy-associated cell death [12,13]. Apoptosis generally occurs due to ROS damage in the mitochondria/endoplasmic reticulum, compared with necrosis due to damage of the plasma membrane or lysosomes [18]. Achieving appropriate ROS and temperature increase levels depends on multiple factors related to the photoresponsive nanoagent (e.g., photothermal conversion efficiency, singlet oxygen quantum yield, dose at the target site) and to laser light [19,20,21,22]. For instance, increasing the laser power density and total exposure time (by augmenting the irradiation time or applying multiple irradiation sessions) can be used to boost the attained photothermal and photodynamic effects [23,24,25,26]. Synchronizing the timepoint for the laser irradiation with the moment when nanoparticles (NPs) achieve their maximum tumor uptake can also be used to improve the therapeutic outcome [27,28,29].

These photo-triggered effects not only induced direct damage on the cancer cells at the primary site but can also (i) relieve tumor hypoxia [30,31,32], (ii) release tumor-associated antigens (TAAs) and danger-associated molecular patterns (DAMPs) [32,33,34], and (iii) induced a pro-inflammatory response [33,35,36]. These events will then synergize with the activity of immunostimulants (toll-like receptor (TLR) agonists) and immune checkpoint inhibitors (ICIs; e.g., IDO1 inhibitors, CTLA-4 and PD-1/PD-L1 blockers) [37,38,39]. Such synergy can pave the way for strong T cell responses against the primary tumor as well as abscopal effects on metastasized cancer cells and the creation of antitumoral immune memory [37,38].

Over the years, different types of nanomaterials with the potential to mediate such photothermal/photodynamic effects have been developed: gold nanospheres [40], gold nanorods [41], platinum NPs [42,43], and polydopamine (PDA) NPs [44,45]. Alternatively, hydrophobic small-molecules with photothermal/photodynamic capabilities (e.g., zinc(II) phthalocyanines [46,47], BODIPY™ [48,49,50], chlorin e6 [51,52,53]) have also been encapsulated in nanomaterials in order to enhance their solubility, cytocompatibility, and/or tumor uptake. Ideally, the nanostructures are exposed to laser light with a wavelength that is similar to the wavelength of the nanostructures’ maximum absorption. For instance, chlorin e6-loaded nanomaterials (λ_max_ = 669 nm) are often irradiated with 660 nm light [54,55,56]. However, the wavelength of the laser light also plays a crucial role in nanomaterials’ phototherapies. Considering that, light in the first near-infrared window (750–1000 nm; henceforward abbreviated as NIR) has a high penetration depth and minimal off-target interactions with biological constituents (e.g., water, melanin, hemoglobin), it is highly desirable to use nanomaterials with a strong NIR light absorption in this therapeutic modality [10].

**Figure 1:**
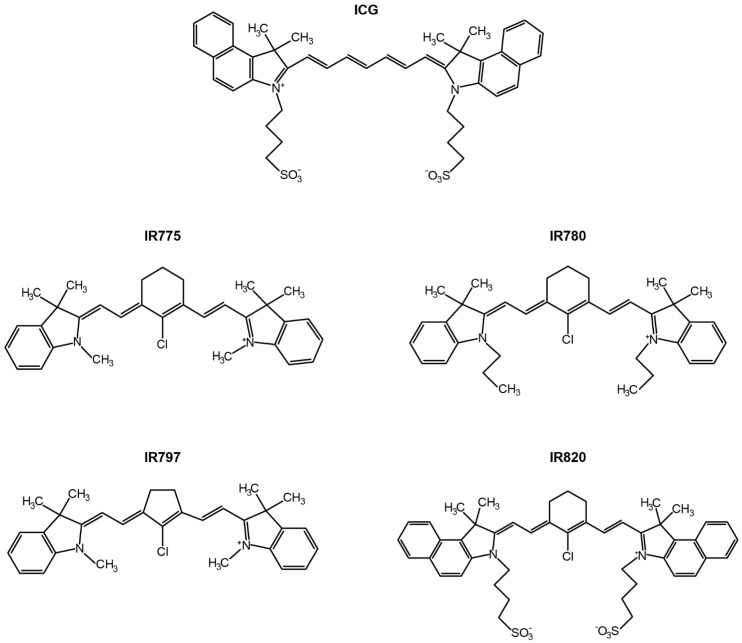
Chemical structure of the HCs (ICG, IR775, IR780, IR797, and IR820) that have been encapsulated in nanomaterials for application in cancer immuno-PTT/PDT.

Among the different NIR light-responsive nanomaterials (e.g., graphene derivatives [57,58], gold nanorods [59,60]), those incorporating heptamethine cyanines (HCs) have received great interest [61,62,63,64,65,66]. These nanostructures can be simply prepared by loading the HCs into the hydrophobic reservoirs of the nanomaterials [61,67]. Moreover, HC-loaded nanomaterials also have a multifunctional character due to the HCs’ photothermal, photodynamic, and fluorescent properties—reviewed in detail by us and by Wang’s group [10,17,68]. In this regard, the FDA-approval status of the HC Indocyanine Green (ICG) has strongly contributed to the investigation of ICG-loaded nanomaterials for cancer immuno-PTT/PDT [69,70]. In addition to ICG = loaded nanomaterials, nanostructures incorporating IR780, IR775, IR797, and IR820 (i.e., prototypic HCs, Figure 1) have also demonstrated strong immuno-PTT/PDT performance due to their superior optical properties [37,71,72].

In this review, the application of HC-loaded nanomaterials in cancer immuno-PTT/PDT is analyzed. In Section 2, a general overview of this therapeutic approach is given. Section 3 and Section 4 analyze the application of ICG-loaded nanomaterials and proto-typic HC-loaded nanostructures in cancer immuno-PTT/PDT. Finally, an outlook of the state of the art and future directions is provided (Section 5).

## 2. Overview of Nanomaterial-Mediated Immuno-PTT/PDT

The photothermal and photodynamic effects mediated by HC-loaded nanomaterials can trigger a series of events that are crucial for potentiating the antitumoral immune responses [31,33,35]. For this reason, the immuno-PTT/PDT potential of these nanomaterials is being investigated for metastatic cancer treatment [32,37,73,74].

In general, this therapeutic approach starts with the intravenous administration of the nanomaterials [75,76]. Once in circulation, these nanomaterials must avoid interaction with blood components (e.g., albumin, red blood cells), uptake by liver/spleen, and rapid clearance by the kidneys [10,77,78]. This will likely increase the probability of these nanomaterials to extravasate to the tumor zone by taking advantage of abnormal static and dynamic pores occurring in the tumor vasculature [68,79,80]. The ability of nanomaterials to avoid off-target accumulation/clearance and to accumulate in the tumor zone is strongly influenced by their physicochemical properties (e.g., size, surface charge, corona composition). The impact of these features on nanomaterial biodistribution has been extensively reviewed by our and other research groups [10,81,82,83,84].

**Figure 2:**
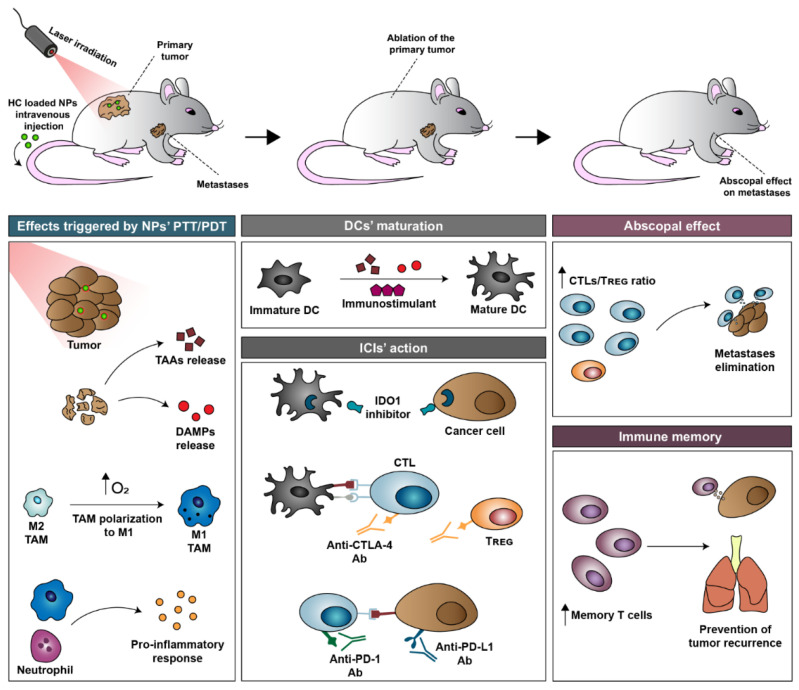
Schematic representation of the different events occurring during the immuno-PTT/PDT mediated by HC-loaded nanoparticles. In this process, the nanoparticles are generally administered intravenously. The immunostimulants and the ICIs can be administered in conjugation with the nanoparticles or at a later time point. After nanoparticle administration, the primary tumor is irradiated with NIR light. The nanoparticles’ photothermal/photodynamic effects can per se induced damage in the primary tumor and can also trigger (i) TAAs/DAMPs release, (ii) hypoxia relief, and (iii) a pro-inflammatory response. The released TAAs can then be processed, leading to DC maturation. DC maturation is also aided by the DAMPs and by the immunostimulants. Afterward, the ICIs abolish the immuno-suppression mediated by CTLA-4, IDO1, and PD-1/PD-L1. These events contribute to the amelioration of the CTL/T_reg_ cells ratio in the diseased sites, paving the way for the elimination of the primary (local effect) and the metastases/distant tumors (abscopal effect). During this process, memory T cells are also established, which have a crucial role in preventing tumor recurrence.

Afterward, the primary tumor (i.e., the original tumor) is irradiated with NIR light, and the nanomaterials accumulated in this zone produce a localized temperature increase and/or ROS [32,71,85]. Such effects can damage cancer cells and may be sufficiently strong to ablate the primary tumor [32,86,87]. As importantly, the nanomaterials’ photothermal/photodynamic effects can also (i) relieve tumor hypoxia [31,32], (ii) release TAAs and DAMPs (e.g., exposure of calreticulin (CRT) on cancer cells’ membrane, release ATP and high mobility group box 1 protein (HMGB1)) [33,34], and (iii) induced a pro-inflammatory response (Figure 2) [35,36].

**Figure 3:**
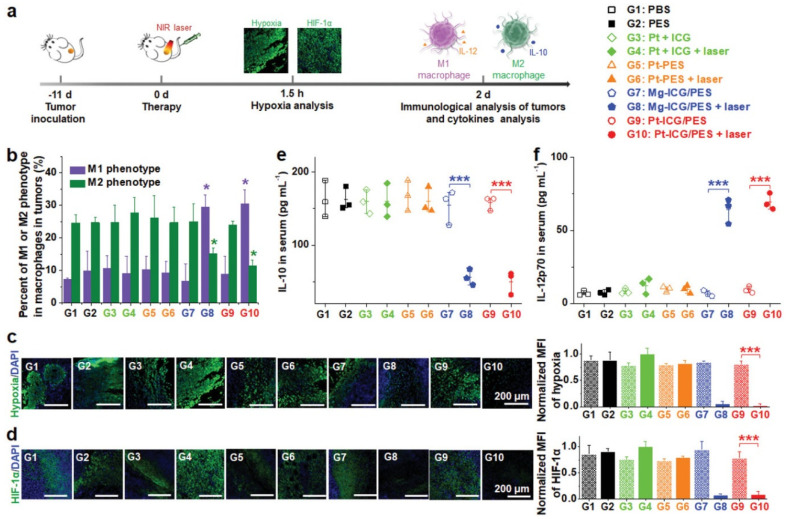
Hypoxia relief triggered by ICG-incorporating polymeric nanostructures when exposed to NIR light. (**a**) Schematic representation of the therapeutic procedure; (**b**) Macrophage phenotype in the tumor after the different treatments; Characterization of the (**c**) hypoxia and (**d**) hypoxia-inducible factor 1-α (HIF-1α) in tumor slices; Levels of (**e**) IL-10 and (**f**) IL-12p70 in the serum of mice after different treatments. Reprinted with permission from [32]. Copyright 2021 Wiley. PBS: phosphate-buffered saline; PES: poly [2-(2-methoxyethoxy) ethyl methacrylate-*co*-PEG methyl ether methacrylate]-*b*-poly(sodium *p*-styrenesulfonate); Pt + ICG: Cisplatin and ICG; Pt-PES: Cisplatin-loaded PES nanoparticles; Mg-ICG/PES: Magnesium dichloride and ICG-loaded PES nanoparticles/ICG-incorporated polymeric nanostructures; Pt-ICG/PES: Cisplatin and ICG-loaded PES nanoparticles; Laser: NIR light exposure. * *p*< 0.05; *** *p*< 0.001.

For instance, Zhao and co-workers demonstrated that PTT/PDT generated by ICG-incorporating polymeric nanostructures induced CRT exposure and HMGB1 and ATP release, leading to about 2.20-fold higher dendritic cell (DC) maturation (when compared to the non-irradiated nanostructures and the control group) [32]. Moreover, the photothermal heating produced by these nanostructures also improved tumor oxygenation. This contributed to augment the tumor levels of M1-polarized (pro-inflammatory/antitumoral) tumor-associated macrophages (TAMs) by 4.30-fold and to reduce the levels of M2-polarized (anti-inflammatory/protumoral) TAMs by 1.70-fold (Figure 3). Tumor hypoxia relief can also be attained or improved through the inclusion of oxygen-generating elements in the nanoformulations (e.g., CeO_2_ nanoparticles [88], MnO_2_ nanoparticles [34], catalase [89]). Tan and co-workers demonstrated that the PTT mediated by cationic lipidic nanoparticles incorporating IR780 could induced the release of TAAs and HMGB1 as well as the exposure of CRT, leading to enhanced DC maturation [33].

These nanomaterials’ photothermal/photodynamic effects can also trigger the release of pro-inflammatory cytokines and chemokines [33,90], which are crucial in the recruitment/activation of immune cells and can also enhance the outcome of ICI-based therapies [12,91,92,93,94].

To further improve DC maturation, immunostimulants can be combined with the HC-loaded nanoparticles. In this regard, CpG oligodeoxynucleotides (CpG ODNs; TLR-9 agonist), due to their hydrophilicity, can be co-administered with the nanoparticles (i.e., non-encapsulated) or incorporated in the hydrophilic shell of the HC-loaded nanoparticles [95,96,97,98]. In turn, hydrophobic immunostimulants such as R837 (Imiquimod; TLR-7 agonist) have been encapsulated in the HC-loaded nanostructures due to their hydrophobicity [69,99]. For example, Chen et al. demonstrated that the ability of the PTT mediated by ICG-loaded poly(lactic-*co*-glycolic acid) (PLGA) nanoparticles to improve DC maturation could be further boosted by 1.24 times by including R837 in this nanoformulation [38].

Subsequently, mature DCs (mDCs) can migrate into the lymph nodes and then prime T cells for the TAA [100,101]. Despite these events, the immunosuppressive actions mediated by CTLA-4, IDO1, and PD-1/PD-L1 can still abrogate the T cells’ action on the primary and secondary tumors [102,103,104,105]. To overcome this bottleneck, ICIs have also been combined with nanomaterial-mediated PTT/PDT [38,75]. In this regard, anti-CTLA-4, anti-PD-1, and anti-PD-L1 antibodies (Ab) are often intravenously co-administered with the nanomaterials (i.e., non-encapsulated) for performing the blockade of these receptors [38,73]. In turn, the IDO1 inhibitors, such as NLG919 or Epacadostat, due to their hydrophobic character, have been-loaded into nanomaterials’ core/reservoirs [85,106]. In general, the combination of ICIs’ action with nanomaterial-mediated PTT/PDT can starkly augment the cytotoxic T lymphocytes (CTLs) populations in the tumoral sites and diminish the populations of regulatory T cells (T_reg_ cells; immunosuppressive cells) [75,85,107], enabling the elimination of the primary tumor and abscopal effects on the secondary tumors (reviewed in Section 3 and Section 4). The combined effects arising from nanomaterial-mediated PTT/PDT and ICIs can also greatly increase the levels of memory T cells [37,38,75], which have a crucial role in decreasing the likelihood of tumor recurrence (reviewed in Section 3 and Section 4).

## 3. ICG-Loaded Nanomaterials in Cancer Immuno-PTT/PDT

ICG-loaded nanomaterials are among the most explored for cancer-immuno-PTT/PDT [107,108,109,110]. The FDA-approval status of ICG for angiography is certainly a key contributor to this phenomenon. The ICG-loaded nanomaterials can be used for theragnostic applications since these can produce a photothermal/photodynamic effect upon NIR laser irradiation as well as emit fluorescence [111,112].

In recent work, Huang et al. verified that the PTT mediated by ICG-loaded Poly(ethylene glycol) functionalized (PEGylated) liposomes could ablate the primary tumor and enrich the CTL/T_reg_ cells ratio in the secondary tumors by 3.30-fold (when compared to the control) [113]. However, such effect was not able to impact the growth of the secondary tumors, which was attributed to the high expression of PD-1 and mucin domain-containing protein 3 (TIM-3) by the secondary tumor-homed CTLs. By combining the nanomaterials’ PTT with PD-1 and TIM-3 blockade (using anti-PD-1 and anti-TIM-3 Abs), secondary tumor regression was attained.

**Figure 4:**
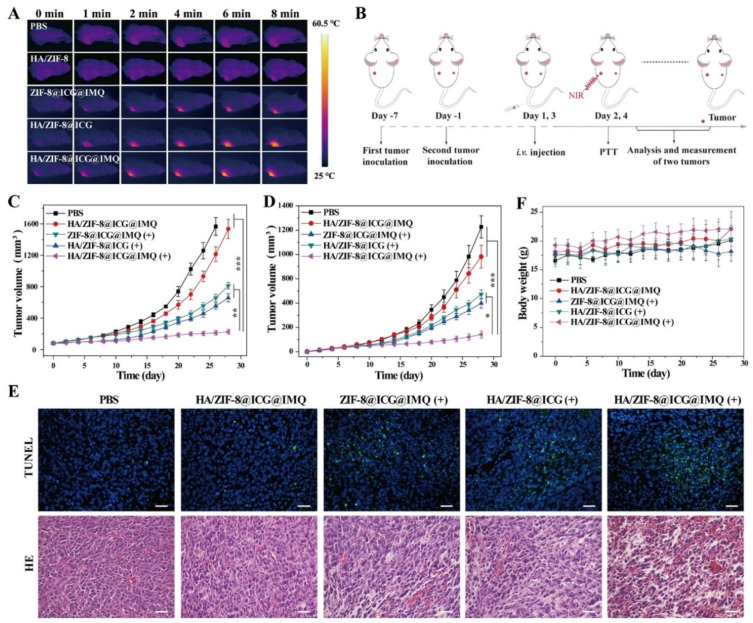
Immuno-PTT mediated by HA-coated MOF-loaded with ICG and R837. (**A**) Mice’s thermal images after NIR irradiation of the primary tumor; (**B**) Schematic representation of the immuno-PTT treatment; (**C**) Primary and (**D**) Secondary tumor volumes after the different treatments; (**E**) TUNEL and hematoxylin and eosin (HE) staining of primary tumor’s slices; Scale bar, 50 μm. (**F**) Mice’s body weight. Reprinted with permission from [108]. Copyright 2020 American Chemical Society. HA/ZIF-8: HA functionalized Zeolitic Imidazolate Framework-8 (ZIF-8) nanoparticles/HA-coated MOF; ZIF-8@ICG@IMQ: ICG- and R837-loaded ZIF-8 nanoparticles; HA/ZIF-8@ICG: ICG-loaded HA functionalized ZIF-8 nanoparticles; HA/ZIF-8@ICG@IMQ: ICG- and R837-loaded HA functionalized ZIF-8 nanoparticles; PTT/(+): NIR light irradiation. * *p* < 0.05, ** *p* < 0.01, *** *p* < 0.001.

In fact, the combination of ICG-loaded nanomaterials’ PTT/PDT capacity with immunostimulants and/or ICIs can pave the way to a remarkable therapeutic outcome [38,39,85,108]. For instance, Liu’s team prepared hyaluronic acid (HA)-coated metal organic frameworks (MOF)-loaded with ICG and R837 for application in cancer immuno-PTT [108]. The combined photothermal and immunostimulatory effects mediated by this nanosystem boosted the levels of mDCs in the lymph nodes to ≈55%, being 1.40 times greater than those attained after the sole application of nanomaterials’ PTT (ICG-loaded MOF plus NIR light) and nanomaterials’ immunostimulant delivery (ICG and R837-loaded MOF) [108]. Due to this reason, the nanomaterial-mediated PTT and R837 delivery induced 1.50 times higher CTL infiltration, thus leading to the greatest reduction in primary and distant tumor growth (Figure 4). This treatment also prompted the highest levels of memory T cells, being the only therapeutic regimen that diminished the growth of the reinoculated tumors.

**Figure 5:**
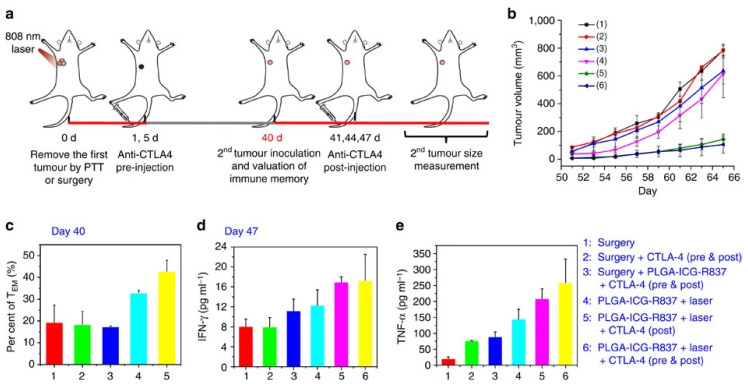
Ability of the immuno-PTT mediated by ICG- and R837-loaded nanoparticles combined with CTLA-4 blockade to prevent tumor recurrence. (**a**) Schematic representation of the treatment procedure; (**b**) Volume of the re-inoculated tumors; (**c**) T_EM_ cells levels in the spleen prior to cancer cell re-inoculation; (**d**) IFN-γ and (**e**) TNF-α levels in the serum of mice seven days after tumor re-inoculation. Reprinted with permission from [38]. Copyright 2016 Nature. Surgery: primary tumor removed by surgery; PLGA-ICG-R837: R837- and ICG-loaded PLGA nanoparticles/PLGA nanoparticles-loaded with ICG and R837; CLTA-4: anti-CTLA-4 Ab administration Laser: NIR light irradiation.

In another study, Chen et al., explored the therapeutic capacity of nanoparticle-mediated PTT and R837 delivery (PLGA nanoparticles-loaded with ICG and R837 plus NIR light) followed by CTLA-4 blockade (systemic administration of anti-CTLA-4 Ab after the PTT) [38]. This combined treatment induced a remarkable effect since it could eliminate the primary and secondary tumors as well as prevent the establishment of metastases. A key contributor to this outcome was the combined treatment’s ability to improve the CTLs/T_reg_ cell ratio in the malignant tissue. In fact, the nanoparticle-mediated PTT and R837 delivery plus CTLA-4 blockade prompted a 1.40, 15.40, 10.20, and 5.60-fold higher CTLs/T_reg_ cell ratio than nanomaterials’ R837 delivery plus CTLA-4 blockade, nanomaterials’ PTT, nanomaterials’ R837 delivery, and CTLA-4 Ab administration, respectively. On other hand, the combined treatment also prompted the highest levels of effector memory T cells (T_EM_), which delayed the growth of the reinoculated tumors (Figure 5). The remaining treatments did not have a meaningful impact on tumor recurrence.

Liu and co-workers prepared PEGylated nanoparticles containing ICG and Epacadostat for cancer immuno-PTT/PDT [85]. The events triggered by the nanoparticles’ photothermal/photodynamic effects could improve DC maturation by up to 2.50-fold (the levels of mDCs in the tumor-draining lymph node (TDLN) reached 16% after nanomaterial- mediated PTT/PDT, contrasting with the 6.4% attained when non-irradiated nanoparticles were used). The nanomaterials’ PTT/PDT combined with IDO1 inhibition (performed by Epacadostat) was able to induced the elimination of the primary tumor and slow the growth of the secondary tumor. By adding PD-L1 blockers to this therapy (PEGylated nanoparticles containing ICG and Epacadostat + NIR light + Anti-PD-L1 Ab), the primary tumor was also eliminated, but the secondary tumor experienced a stronger delay in its growth. Such events were correlated with a higher CTL infiltration and higher amelioration of the CTL/T_reg_ cells ratio in the secondary tumors after the nanomaterials’ PTT/PDT combined with IDO1 inhibition and PD-L1 blockade. In other work, Lam’s team demonstrated that the application of two treatment cycles composed of R837-loaded PEGylated ICG-based nanoparticles plus NIR light plus anti-PD-1 Ab administration could lead to the elimination of both primary and secondary tumors [39].

The immuno-PTT/PDT capability of other ICG-loaded nanomaterials is summarized in Table 1 and Table 2.

## 4. Prototypic HC-Loaded Nanomaterials in Cancer Immuno-PTT/PDT

Nanoparticles containing prototypic HC also hold great potential for application in cancer immuno-PTT/PDT due to their improved optical properties (reviewed in detail in [10,17,68]). Among these, IR780-loaded nanomaterials have been the most applied, followed by IR820-loaded nanostructures.

As described in Section 2, the events triggered by the nanomaterial-mediated PTT/PDT can per se support the development of antitumoral immunological responses. In this regard, Borrathybay and co-workers verified that the photothermal/photodynamic effects generated by IR780-loaded PEG-Poly(caprolactone) (PCL) nanoparticles trigger the release of DAMPs (ATP, HMGB1, CRT), leading to a 1.50- and 2-fold greater DCs’ maturation and CTLs’ infiltration when compared to the control, respectively [122]. These effects paved the way for a slight decrease in the primary tumors’ growth and reduction of the occurrence of lung metastases.

**Figure 6:**
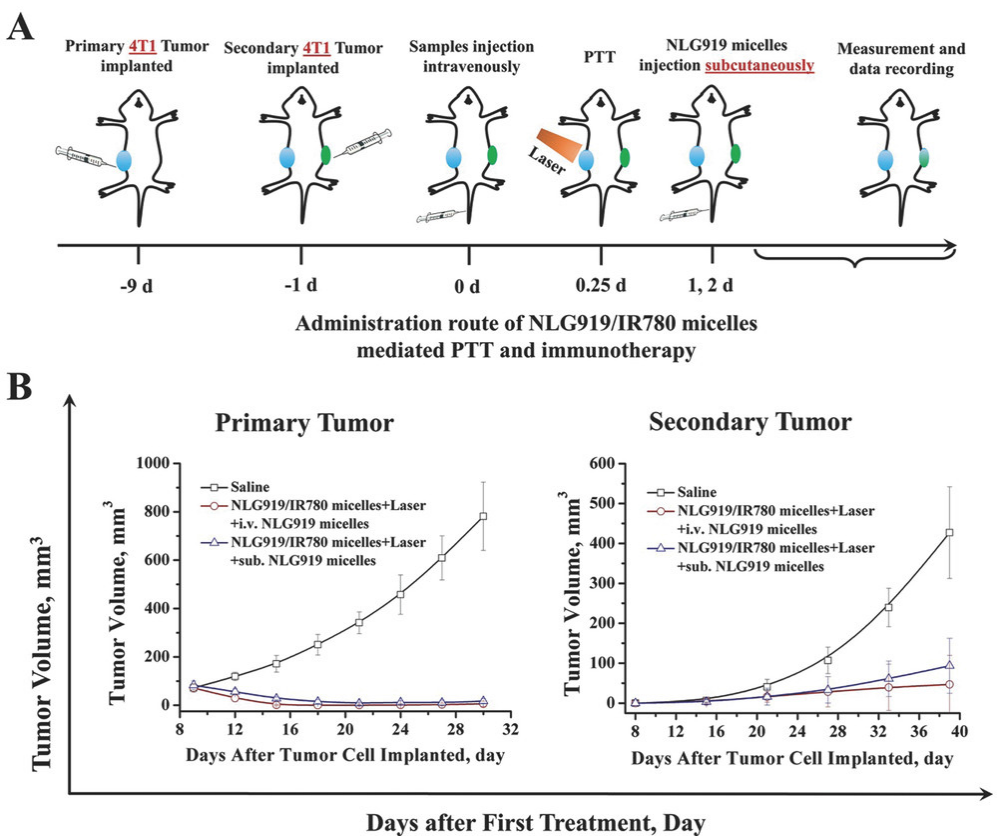
Immuno-PTT mediated by PEG-PCL micelles-loaded with NLG919 and IR780 in primary and secondary tumors. (**A**) Schematic representation of the treatment schedule; (**B**) Primary and (**B**) Secondary tumor volumes after different treatments. Reprinted with permission from [106]. Copyright 2018 Wiley. NLG919/IR780 micelles: PEG-PCL micelles-loaded with NLG919 and IR780; NLG919 micelles: PEG-PCL micelles-loaded with NLG919; i.v.: intravenous administration; sub.: subcutaneous administration; Laser: NIR light irradiation.

The inclusion of immunostimulants and/or ICIs in the nanomaterials’ phototherapies is crucial to further boost the therapeutic outcome [37,65,71,75,106]. For instance, Ou and co-workers prepared PEGylated Glucocorticoid-induced Cancer Necrosis Factor Receptor (GITR)-functionalized PLGA nanoparticles incorporating IR780 and Imatinib (diminishes immunosuppression mediated by T_reg_ cells [123]) for application in cancer immuno-PTT/PDT [71]. The irradiation of these nanoparticles with NIR light stimulated the release of TAAs and HMBG1. This could augment the intratumoral levels of matured DCs to about 52%, being 2.40 times greater than those attained in the control group [71]. Moreover, the IR780 and Imatinib-loaded nanoparticles combined with NIR light also reduced the intratumoral T_reg_ cells’ levels by 3.40-fold. Such events mediated by the nanomaterials’ immuno-PTT/PDT led to complete tumor elimination. In another work, Qian et al., developed PEG-PCL micelles-loaded with NLG919 and IR780 for application in cancer immuno-PTT [106]. By combining the IDO1 inhibitory capacity of NLG919 with the local hyperthermia produced by the micelles upon NIR laser irradiation, this treatment could ablate the primary tumor and strongly diminish the growth of the secondary tumors (Figure 6). Moreover, this combined approach also decreased the establishment of lung metastases. This outcome was correlated with the ability of the micelle-mediated immuno-PTT to greatly improve the CTLs/T_reg_ cells ratio. In fact, the micelles’ immuno-PTT prompted a 7- and 33 times higher CTLs/T_reg_ cells ratio than the micelles’ immunotherapy (NLG919 and IR780-loaded micelles) and micelles’ PTT (IR780-loaded micelles plus NIR light), respectively. Therefore, the micelles’ immunotherapy and micelles’ PTT were only capable of reducing the growth of the primary and secondary tumors.

In another work, Luan team prepared HA-coated IR820-loaded MOFs and Mannan-coated R837 and 1-Methyl-D-tryptophan (1MT; IDO1 inhibitor)-loaded MOFs for cancer immuno-PTT. The photothermal effect mediated by the IR820-loaded MOFs strongly stimulated DAMPs and TAAs release [37]. Such effect could improve DCs’ maturation levels from 17.6 to 33.2%. By combining the PTT capacity of IR820-loaded MOFs with the immunomodulating capacity of R837 and 1MT-loaded MOFs, the levels of mDCs could be further improved to about 42%. In vivo, the combined treatment (IR820-loaded MOFs + NIR light + R837 and 1MT-loaded MOFs) prompted the greatest enrichment in the CTL/T_reg_ cells ratio. Such events contributed to the regression of the primary tumor and almost inhibited the growth of the secondary tumor. This combined treatment could also abolish the establishment of metastases upon reinoculation of the cancer cells, an effect attributed to the more pronounced presence of memory T cells.

**Figure 7:**
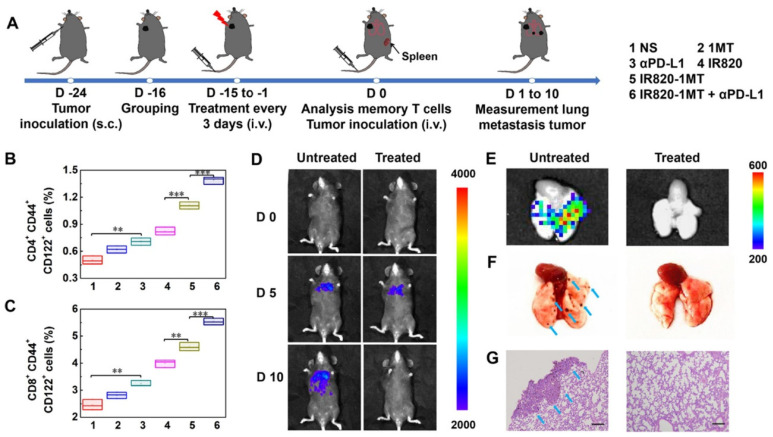
Ability of the immuno-PTT mediated by IR820-1MT conjugate nanoparticles in combination with PD-L1 blockade to prevent tumor recurrence in the lungs. (**A**) Schematic representation of the therapeutic approach; (**B**,**C**) Levels of T_EM_ cells in the spleen after the different treatments; (**D**) Bioluminescence images of lung metastases in mice overtime; (**E**) Ex vivo bioluminescence and (**F**) optical images of the lungs; (**G**) HE staining of the lung slices. Reprinted with permission from [75]. Copyright 2019 American Chemical Society. NS: normal saline; αPD-L1: anti-PD-L1 Ab; IR820: IR820 + Laser irradiation; IR820-1MT: IR820-1MT conjugate nanoparticles + Laser irradiation. ** *p* < 0.01, and *** *p* < 0.001. The blue arrows represent metastatic nodules.

Luan’s team explored the phototherapeutic potential of IR820-1MT conjugate nanoparticles in combination with Anti-PD-L1 Ab [75]. Pairing the double-ICI strategy with the PTT (IR820-1MT + NIR light + Anti-PD-L1 Ab) yielded the best therapeutic outcome (the strongest reduction in the growth of the primary and secondary tumors), due to greater DCs’ maturation, CTLs’ infiltration, and CTL/T_reg_ ratio improvement. This approach also prompted the highest levels of T_EM_ cells, and therefore the establishment of lung metastases did not occur after tumor reinoculation (Figure 7).

The immuno-PTT/PDT potential of other prototypic HC-loaded nanomaterials is summarized in Table 3 and Table 4.

## 5. Conclusions and Future Outlook

In this review, the recent progress in the application of HC-loaded nanomaterials for cancer immuno-PTT/PDT was analyzed.

Among the HC family, the ICG-loaded nanomaterials have been the most explored for this therapeutic modality, followed by those-loaded with IR780 and then by those incorporating IR820, IR797, and IR775. This trend is concomitant with the usage of these nanostructures in other areas (e.g., standalone PTT/PDT, chemo-PTT/PDT). On the one hand, the FDA-approved status of ICG has fomented the development of nanomaterials containing this NIR dye for cancer-related applications. On the other hand, prototypic HCs such as IR780 and IR820 have superior optical properties when compared to ICG, which has motivated their loading into nanomaterials aimed for cancer therapy. Despite the potential of other prototypic HCs (e.g., Cypate, IR808, IR825), these have not yet been explored for cancer immuno-PTT/PDT. Therefore, in the future, the development of nanoformulations incorporating such prototypic HCs could be interesting to fully unveil their immuno-phototherapeutic potential.

In general, the coordinated action of HC-loaded nanomaterials’ photothermal/photodynamic effects (e.g., inducers of cell death and release of TAAs/DAMPs), immunostimulants (enhancers of DCs’ maturation), and ICIs (strong modulators of CTLs and T_reg_ cells) could elicit both local (on the primary tumor) and abscopal (on the secondary tumor/metastases) antitumoral responses. In some few cases, the magnitude of such combined effects led to the complete elimination of the primary tumor and also induced a reduction in the growth of the secondary tumor or even its elimination. These combined immuno-phototherapeutic effects also had an important role in the establishment of immune memory that could prevent/delay tumor’s recurrence. Together, these facts depict the potential of HC-loaded nanomaterials for cancer immuno-PTT/PDT.

In order to further amplify the magnitude of HC-loaded nanomaterials’ immuno-PTT/PDT, it could be interesting to (i) boost HC-loaded nanomaterials’ photothermal/photodynamic capacity, (ii) optimize the delivery regiment of immunostimulants/ICIs, and (iii) incorporate additional therapeutic agents in the combined therapy. Boosting the HC-loaded nanomaterials’ photothermal/photodynamic effects will be crucial to enhance the therapeutic outcome in the primary tumor as well as to potentially improve the release of DAMPs/TAAs, which play an important role in the abscopal antitumoral T cell responses. This could be achieved by improving HC-loaded nanomaterials’ photostability (in order to sustain the phototherapeutic effects over time) or by incorporating additional NIR responsive agents in the nano-formulations (e.g., gold nanorods, graphene derivatives) [125,126,127].

The events occurring in HC-loaded nanomaterials’ immuno-PTT/PDT set the optimal time points for the action of the immunostimulants and ICIs. Initially, the nanomaterials’ photothermal/photodynamic effects must occur to trigger TAAs/DAMPs release, which will be crucial for DC maturation. In this way, the immunostimulants’ action is best suited after the nanomaterials’ PTT/PDT. The same applies to ICIs, whose action is optimal after DC maturation. Therefore, the development of technologies that can perform the sequential delivery of nanomaterials, immunostimulants, and ICIs can potentially pave the way for an improved therapeutic outcome. In this context, hierarchically designed injectable hydrogels, microneedle patches, and scaffolds are promising tri-dimensional matrixes for performing the sequential delivery of these agents [128,129,130,131]. Finally, the inclusion of other hydrophobic agents in the nanomaterials’ core/reservoirs (e.g., chemotherapeutic drugs) or hydrophilic agents in the abovementioned tri-dimensional *matrices* (e.g., antitumoral peptides) can lead to an even greater therapeutic outcome by exploring synergistic interactions among the enrolled agents [9,96,132,133,134].

Despite the potential of HC-loaded nanomaterials’ immuno-PTT/PDT, this therapeutic approach still faces critical challenges before validation in clinical trials can be envisioned. So far, HC-loaded nanomaterials’ immuno-PTT/PDT has been mainly applied to treat breast and melanoma tumors and their metastases in mice. This applicability to superficial tumors is highly correlated with the penetration depth limits of NIR light [135,136]. Furthermore, human tumors are also located in deeper zones when compared to their equivalents in mice [137]. In this regard, the use of endoscopes coupled with fiber-type laser to irradiate deeper primary tumors may be an interesting strategy to address the previous limitations at the cost of increasing the procedures’ invasiveness [137].

There are also hurdles associated with the nanomaterials’ tumor-homing capacity. Classically, nanomaterials have been described to accumulate in the tumor by extravasating through the tumor’s leaky vasculature (the so-called enhanced permeability and retention (EPR) effect), hence being designed based on this rationale. However, a review by Wilhelm et al., highlighted that the dose of intravenously administered nanoparticles that reaches the tumor is, in many cases/studies, very low [138]. Recently, other mechanisms involved in nanomaterials’ tumor accumulation have been unveiled (e.g., dynamic vents, active transport through endothelial cells) [79,139]. In this way, it is crucial to continue to investigate the mechanisms responsible for nanomaterials’ tumor uptake after systemic administration and to optimize the nanoparticles’ physicochemical properties accordingly. Moreover, strategies aimed to modulate the tumor vasculature could be a route for mitigating this tumor uptake problem (e.g., vascular permeabilization, normalization, or disruption approaches) [140,141,142,143]. On the other hand, the encapsulation of nanomaterials in macroscale delivery systems (e.g., injectable hydrogels, microneedles) is also appealing [130,131]. These macroscale systems can be used to perform the direct delivery of nanoparticles and ICIs/immunostimulants into the tumor site, possibly avoiding the abovementioned systemic administration issues [131,144].

On the other hand, the efficacy of HC-loaded nanomaterials’ immuno-PTT/PDT has not yet been validated in larger animal models. These studies are of utmost importance since they can expose some of the limitations described above. Moreover, long-term studies are also required. Such studies are crucial to establish the safety of this approach since some possible side effects may have a delayed onset (e.g., immune-related adverse events) [145]. Moreover, the outcome of nanomaterial-mediated immuno-PTT/PDT can be, in some cases, highly heterogenous (the same also occurs in the clinic for ICIs) [106,107,110]. In this regard, finding biomarkers that can predict the therapeutic response may also be a path to push the translation of this strategy [146].

Overall, continuing this line of research based on HC-loaded nanomaterials’ immuno-PTT/PDT can unlock potent antitumoral T cell responses against local and metastasized cancer cells as well as generate immune memory that prevents tumor’s recurrence.

## Figures and Tables

**Table 1:** Outcome generated by the immuno-PTT/PDT mediated by ICG-based nanomaterials in the levels of mDCs and T cells.

Formulation	Immuno Therapy Agent	PTT/ PDT Agent	Changes in the Levels of mDCs and T Cells	Ref
**R837- and ICG-loaded PLGA NPs**	R837; Anti-CTLA-4 Ab (non-loaded)	ICG	R837- and ICG-loaded PLGA NPs + Laser induced 1.24 and 1.32 times higher mDC levels than the respective ICG-loaded PLGA NPs + Laser and R837- and ICG-loaded PLGA NPs (in the tumor-draining lymph nodes).	[38]
			R837- and ICG-loaded PLGA NPs + Laser + anti-CTLA-4 Ab induced 1.40, 15.42, 10.15, 5.63, and 3.05 times higher CTLs/T_reg_ ratios than surgery + R837- and ICG-loaded PLGA NPs + anti-CTLA-4 Ab, ICG-loaded PLGA NPs + Laser, surgery + R837- and ICG-loaded PLGA NPs, surgery + anti-CTLA-4 Ab, and surgery, respectively (in the secondary tumor).	
			R837- and ICG-loaded PLGA NPs + Laser + anti-CTLA-4 Ab (post tumor reinoculation) resulted in 1.31, 2.50, 2.26, and 2.24 times higher T_EM_ cells levels than R837- and ICG-loaded PLGA NPs + Laser, surgery + R837 and ICG-loaded PLGA NPs + anti-CTLA-4 Ab (pre and post tumor reinoculation), surgery + anti-CTLA-4 Ab (pre and post tumor reinoculation), and surgery, respectively (in the spleen).	
**ICG and RB ^(a)^-loaded DSPE ^(b)^-PEG-mal ^(c)^ functionalized UCNPs ^(d)^**	-	ICG; RB	ICG- and RB-loaded DSPE-PEG-mal functionalized UCNPs + Laser induced 1.94 and 3.02 times higher mDC levels than ICG- and RB-loaded DSPE-PEG functionalized UCNPs + Laser, and the control + Laser, respectively (in the primary tumor).	[114]
			ICG- and RB-loaded DSPE-PEG-mal functionalized UCNPs + Laser induced 3.10 and 5.69 times higher CTLs/T_reg_ ratios than ICG- and RB-loaded DSPE-PEG functionalized UCNPs + Laser, and the control + Laser, respectively (in the secondary tumor); ICG- and RB-loaded DSPE-PEG-mal functionalized UCNPs + Laser induced 1.14 and 1.46 times higher CTLs levels than ICG- and RB-loaded DSPE-PEG functionalized UCNPs + Laser, and the control + Laser, respectively (in the spleen).	
**Mg and ICG-loaded PES NPs**	-	ICG	Mg and ICG-loaded PES NPs + Laser induced two times higher mDC levels than Mg and ICG-loaded PES NPs, and PES (in the primary tumor); Mg and ICG-loaded PES NPs + Laser induced 2.27 times higher mDC levels than the control (in the primary tumor); Mg and ICG-loaded PES NPs + Laser induced about two times higher mDC levels than Mg and ICG-loaded PES NPs, PES, and the control (in lymph nodes).	[32]
			Mg and ICG-loaded PES NPs + Laser induced about 2.72 times higher CTLs levels than Mg and ICG-loaded PES NPs, PES, and the control (in the secondary tumor).	
**ICG-loaded COF ^(e)^ coated with ovalbumin**	Anti-PD-L1 Ab (non-loaded)	ICG; COF	ICG-loaded COF coated with ovalbumin + Laser + anti-PD-L1 Ab induced 1.31, 1.82, and 2.22 times higher mDC levels than ICG-loaded COF coated with ovalbumin + Laser, PBS + anti-PD-L1 Ab, and the control, respectively (in lymph nodes).	[73]
			ICG-loaded COF coated with ovalbumin + Laser + anti-PD-L1 Ab induced 1.29, 2.05, and 2.51 times higher CTLs levels than ICG-loaded COF coated with ovalbumin + Laser, PBS + anti-PD-L1 Ab, and the control, respectively (in the primary tumor).	
**ICG-loaded liposome ^(f)^**	-	ICG	ICG-loaded liposome + Laser induced 3.29 times higher CTLs/T_reg_ ratios than the control (in the secondary tumor).	[113]
**ICG-loaded PEG-Epacadostat conjugate NPs**	Epacadostat; Anti-PD-L1 Ab (non-loaded)	ICG	ICG-loaded PEG-Epacadostat conjugate NPs + Laser induced 2.47, 2.27, and 3.83 times higher mDC levels than ICG-loaded PEG-Epacadostat conjugate NPs, PEG-Epacadostat conjugate NPs, and the control, respectively (in lymph nodes).	[85]
			ICG-loaded PEG-Epacadostat conjugate NPs + Laser + anti-PD-L1 Ab induced 1.91, 2.10, 8.17, 6.10, and 6.81 times higher CTLs/T_reg_ ratios than ICG-loaded PEG-Epacadostat conjugate NPs + anti-PD-L1 Ab, ICG-loaded PEG-Epacadostat conjugate NPs + Laser, ICG-loaded PEG-Epacadostat conjugate NPs, anti-PD-L1 Ab, and the control, respectively (in the secondary tumor).	
**ICG-loaded lipid ^(g)^-PLGA NPs decorated with FimH ^(h)^**	FimH	ICG	ICG-loaded lipid-PLGA NPs decorated with FimH + Laser, and FimH + Laser induced about three times higher mDC levels than ICG-loaded lipid-PLGA NPs + Laser, lipid-PLGA NPs + Laser, and the control, respectively (in lymph nodes).	[115]
**ICG-loaded PLGA based NPs incorporated into decitabine, DSPE-PEG, and cell membranes based NPs**	Decitabine	ICG	ICG-loaded PLGA based NPs incorporated in decitabine, DSPE-PEG, and cell membrane-based NPs + Laser induced 1.74, 3.28, 15.24, 7.38, and 12.63 times higher mDC levels than ICG-loaded PLGA based decitabine lipidic NPs + Laser, ICG + Decitabine + Laser, Decitabine + Laser, ICG + Laser, and the control, respectively (in the primary tumor); ICG-loaded PLGA-based NPs incorporated in decitabine, DSPE-PEG, and cell membrane-based NPs + Laser induced 1.73, 5.20, 8.27, 10.82, and 12.06 times higher mDC levels than ICG-loaded PLGA based decitabine lipidic NPs + Laser, ICG + Decitabine + Laser, Decitabine + Laser, ICG + Laser, and the control, respectively (in tumor-draining lymph nodes).	[116]
			ICG-loaded PLGA-based NPs incorporated in decitabine, DSPE-PEG, and cell membrane-based NPs + Laser induced 2, 3.86, 6, 4.50, and 4.93 times higher CTLs levels than ICG-loaded PLGA based decitabine lipidic NPs + Laser, ICG + Decitabine + Laser, Decitabine + Laser, ICG + Laser, and the control, respectively (in the secondary tumor); ICG-loaded PLGA-based NPs incorporated in decitabine, DSPE-PEG, and cell membrane-based NPs + Laser induced 1.52, 2.56, 4.27, 3.37, and 5.49 times higher CTLs levels than ICG-loaded PLGA-based decitabine lipidic NPs + Laser, ICG + Decitabine + Laser, Decitabine + Laser, ICG + Laser, and the control, respectively (in the spleen).	
**CAT ^(i)^, DTA-1 ^(j)^ and ICG functionalized PDA ^(k)^ NPs**	CAT	ICG; PDA	CAT, DTA-1, and ICG functionalized PDA NPs + Laser induced 2.17, 2.48, 2.74, and 2.47 times higher mDC levels than ICG-functionalized PDA NPs + Laser, ICG + Laser, PDA + Laser, and the control, respectively (in spleen).	[117]
			CAT, DTA-1, and ICG-functionalized PDA NPs + Laser induced 2.89, 2.03, 2.89, and 3.68 times higher CTLs/T_reg_ ratios than ICG-functionalized PDA NPs + Laser, ICG + Laser, PDA + Laser, and the control, respectively (in the primary tumor).	
**ICG-loaded Mn@CaCO_3_ NPs functionalized with siPD-L1 ^(l)^ and PAH ^(m)^**	siPD-L1	ICG	ICG-loaded Mn@CaCO_3_ NPs functionalized with siPD-L1 and PAH + Laser induced 2.61, 4.50, 5.06, and 9 times higher mDC levels than ICG-loaded Mn@CaCO_3_ NPs + Laser, ICG-loaded Mn@CaCO_3_ NPs functionalized with siPD-L1 and PAH, ICG-loaded Mn@CaCO_3_ NPs, and the control, respectively (in the primary tumor).	[118]
			ICG-loaded Mn@CaCO_3_ NPs functionalized with siPD-L1 and PAH + Laser induced 2, 20, 26.67, and 80 times higher CTLs levels than ICG-loaded Mn@CaCO_3_ NPs + Laser, ICG-loaded Mn@CaCO_3_ NPs functionalized with siPD-L1 and PAH, ICG-loaded Mn@CaCO_3_ NPs, and the control, respectively (in the primary tumor).	
**Anti-PD-L1 Ab and ICG-loaded PEG-PLGLAG ^(n)^-dEGCG ^(o)^ NPs**	Anti-PD-L1 Ab	ICG	Anti-PD-L1 Ab and ICG-loaded PEG-PLGLAG-dEGCG NPs + Laser induced 2.18, 1.25, 2.95, 1.29, 3.25, 2.60 and 3.53 times higher mDC levels than Anti-PD-L1 Ab and ICG-loaded PEG-PLGLAG-dEGCG NPs, Anti-PD-L1 Ab, and ICG-loaded EGCG NPs + Laser, Anti-PD-L1 Ab, and ICG-loaded EGCG NPs, ICG-loaded PEG-PLGLAG-dEGCG NPs + Laser, ICG-loaded PEG-PLGLAG-dEGCG NPs, Anti-PD-L1 Ab, and the control, respectively (in the primary tumor); Anti-PD-L1 Ab and ICG-loaded PEG-PLGLAG-dEGCG NPs + Laser induced 2.15, 1.26, 1.44, 5.68, 2.79, and 7 times higher mDC levels than Anti-PD-L1 Ab and ICG-loaded PEG-PLGLAG-dEGCG NPs, Anti-PD-L1 Ab and ICG-loaded EGCG NPs + Laser, ICG-loaded PEG-PLGLAG-dEGCG NPs + Laser, ICG-loaded PEG-PLGLAG-dEGCG NPs, Anti-PD-L1 Ab, and the control, respectively (in lymph nodes of lymphatic metastases).	[107]
			Anti-PD-L1 Ab and ICG-loaded PEG-PLGLAG-dEGCG NPs + Laser induced 2.77, 2.03, 6.91, 2.54, 10.46, 4.07, and 10.77 times higher CTLs/T_reg_ ratio than Anti-PD-L1 Ab and ICG-loaded PEG-PLGLAG-dEGCG NPs, Anti-PD-L1 Ab, and ICG-loaded EGCG NPs + Laser, Anti-PD-L1 Ab and ICG-loaded EGCG NPs, ICG-loaded PEG-PLGLAG-dEGCG NPs + Laser, ICG-loaded PEG-PLGLAG-dEGCG NPs, Anti-PD-L1 Ab, and the control, respectively (in tumor-infiltrating lymphocytes); Anti-PD-L1 Ab and ICG-loaded PEG-PLGLAG-dEGCG NPs + Laser induced 2.15, 2.67, 2.26, 5.46, 2.84, and 4.40 times higher CTLs levels than Anti-PD-L1 Ab and ICG-loaded PEG-PLGLAG-dEGCG NPs, Anti-PD-L1 Ab, and ICG-loaded EGCG NPs + Laser, ICG-loaded PEG-PLGLAG-dEGCG NPs + Laser, ICG-loaded PEG-PLGLAG-dEGCG NPs, Anti-PD-L1 Ab, and the control, respectively (in lymph nodes).	
**ICG and R837-loaded HA functionalized ZIF-8 NPs**	R837	ICG	ICG- and R837-loaded HA-functionalized ZIF-8 NPs + Laser induced 1.44, 1.35, 1.31, and 1.82 times higher mDC levels than ICG-loaded HA-functionalized ZIF-8 NPs + Laser, ICG and R837-loaded ZIF-8 NPs + Laser, ICG- and R837-loaded HA-functionalized ZIF-8 NPs, and the control, respectively (in lymph nodes).	[108]
			ICG- and R837-loaded HA functionalized ZIF-8 NPs + Laser induced about 1.50 times higher CTLs levels than ICG-loaded HA-functionalized ZIF-8 NPs + Laser, ICG- and R837-loaded ZIF-8 NPs + Laser, and ICG- and R837-loaded HA- functionalized ZIF-8 NPs (in the primary tumor); ICG- and R837-loaded HA-functionalized ZIF-8 NPs + Laser induced 3.11 times higher CTLs levels than control (in the primary tumor).	
			ICG- and R837-loaded HA-functionalized ZIF-8 NPs + Laser generated 1.67, 1.69, 2.27, and 3.45 times higher memory T cells levels than ICG-loaded HA-functionalized ZIF-8 NPs + Laser, ICG- and R837-loaded ZIF-8 NPs + Laser, ICG- and R837--loaded HA-functionalized ZIF-8 NPs, and the control, respectively (in the spleen).	
**ICG and R837-loaded PEG-polyphenols functionalized Fe_3_O_4_ based NPs**	R837	ICG	ICG- and R837-loaded PEG-polyphenol-functionalized Fe_3_O_4_-based NPs + Laser induced 1.37, 1.21, 2.06, and 2.00 times higher mDC levels than R837-loaded PEG-polyphenol-functionalized Fe_3_O_4_-based NPs + Laser, ICG-loaded PEG-polyphenol-functionalized Fe_3_O_4_-based NPs + Laser, PEG-polyphenol-functionalized Fe_3_O_4_-based NPs + Laser, and the control, respectively (in lymph nodes).	[69]
			ICG- and R837-loaded PEG-polyphenol-functionalized Fe_3_O_4_-based NPs + Laser induced about 1.18 times higher CTLs levels than R837-loaded PEG-polyphenol-functionalized Fe_3_O_4_-based NPs + Laser, and ICG-loaded PEG-polyphenol-functionalized Fe_3_O_4_-based NPs + Laser (in the primary tumor); ICG- and R837-loaded PEG-polyphenol-functionalized Fe_3_O_4_-based NPs + Laser induced 1.41 and 4.13 times higher CTLs levels than PEG-polyphenol-functionalized Fe_3_O_4_-based NPs + Laser, and the control, respectively (in the primary tumor).	
**ICG and PM ^(p)^-loaded albumin MnO_2_ NPs**	PM; MnO_2_	ICG	ICG- and PM-loaded albumin MnO_2_ NPs + Laser induced 1.23, 1.93, and 2.73 times higher CTLs levels than ICG- and PM-loaded albumin MnO_2_ NPs, ICG-loaded albumin MnO_2_ NPs + Laser, and the control, respectively (in the primary tumor).	[110]
**FAL ^(q)^-PEG-TA ^(r)^ and PEI ^(s)^-ICG functionalized AuNS ^(t)^ and Hb ^(u)^-loaded FAL liposomes ^(v)^**	Hb	ICG; AuNS	FAL-PEG-TA- and PEI-ICG-functionalized AuNS and Hb-loaded FAL liposomes + Laser induced 1.25, 1.56, 4, 1.73, and 3.76 times higher mDC levels than FAL-PEG-TA- and PEI-ICG-functionalized AuNS + Laser, PEI-ICG-functionalized AuNS + Hb-loaded liposomes + Laser, PEI-ICG-functionalized AuNS + Laser, FAL-PEG-TA-functionalized AuNS + Laser, and the control, respectively (in lymph nodes).	[76]
			FAL-PEG-TA- and PEI-ICG-functionalized AuNS and Hb-loaded FAL liposomes + Laser induced 1.56, 2, 2.19, 1.04, and 2.80 times higher CTLs levels than FAL-PEG-TA- and PEI-ICG-functionalized AuNS + Laser, PEI-ICG-functionalized AuNS + Hb-loaded liposomes + Laser, PEI-ICG-functionalized AuNS + Laser, FAL-PEG-TA-functionalized AuNS + Laser, and the control, respectively (in splenic lymphocytes); FAL-PEG-TA- and PEI-ICG-functionalized AuNS and Hb-loaded FAL liposomes + Laser induced 1.80 and 1.75 times lower T_reg_ levels than PEI-ICG-functionalized AuNS + Hb-loaded liposomes + Laser, and the control, respectively (in spleens).	
**ICG-loaded PLGA NPs incorporated in EPV ^(w)^**	EPV	ICG	ICG-loaded PLGA NPs incorporated in EPV + Laser induced 1.14, 1.22, 1.80, 1.23, 1.59, 1.62, 2, and 2.15 times higher CTLs levels than ICG-loaded PLGA NPs incorporated in Melanoma membrane vesicles + Laser, ICG-loaded PLGA NPs incorporated in Salmonella membrane vesicles + Laser, ICG-loaded PLGA NPs + Laser, ICG-loaded PLGA NPs incorporated in EPV, ICG-loaded PLGA NPs incorporated in Melanoma membrane vesicles, ICG-loaded PLGA NPs incorporated in Salmonella membrane vesicles, ICG-loaded PLGA NPs, and the control, respectively (in the primary tumor).	[119]
**ICG, R837, and CTL-Ap ^(x)^-loaded dextran NPs**	R837; CTL-Ap	ICG	ICG-, R837-, and CTL-Ap-loaded dextran NPs + Laser induced 2.41 times higher CTLs levels than non-irradiated NPs (in the primary tumor).	[109]
**ICG and R837-IONs ^(y)^-loaded DSPE-PEG NPs**	R837; IONs	ICG	ICG- and R837-ION-loaded DSPE-PEG NPs + Laser induced 1.80 times higher CTLs/T_reg_ ratios than ICG- and ION-loaded DSPE-PEG NPs + Laser (in the primary tumor); ICG- and R837-ION-loaded DSPE-PEG NPs + Laser induced about 3.20 times higher CTLs/T_reg_ ratios than ICG- and R837-ION-loaded DSPE-PEG NP-loaded DSPE-PEG NPs, control + Laser, and the control (in the primary tumor).	[99]

^(a)^ Rose Bengal; ^(b)^ 1,2-distearoyl-*sn*-glycero-3-phosphoethanolamine; ^(c)^ Maleimide; ^(d)^ Upconversion NPs; ^(e)^ Covalent Organic Framework; ^(f)^ Formulated with Dipalmitoyl Phosphatidylcholine (DPPC), DSPE-PEG, and cholesterol; ^(g)^ Lecithin and 1,2-dioleoyl-*sn*-glycero-3-[(*N*-(5-amino-1-carboxypentyl)iminodiacetic acid)succinyl] (nickel salt); ^(h)^ *Escherichia coli* Type 1 Fimbriae Adhesion Portion; ^(i)^ Catalase; ^(j)^ Anti-GITR Ab; ^(k)^ Polydopamine; ^(l)^ PD-L1-targeting siRNA; ^(m)^ Polycyclic-aromatic Hydrocarbons; ^(n)^ Proline–leucine–glycine–leucine–alanineglycine; ^(o)^ Epigallocatechin-3-*O*-gallate; ^(p)^ Phenformin; ^(q)^ Endoplasmic Reticulum (ER)-targeting Pardaxin; ^(r)^ Thioctic Acid; ^(s)^ Polyethylenimine; ^(t)^ Gold Nanospheres; ^(u)^ Hemoglobin; ^(v)^ Formulated with Egg phosphatidyl lipid-80, cholesterol, DSPE-PEG, and FAL-DSPE-PEG; ^(w)^ Salmonella–Melanoma Membrane Vesicles; ^(x)^ Cytotoxic T Lymphocyte Antigen Peptide with the sequence SIINFEKL; ^(y)^ Iron Oxide NPs.

**Table 2:** Outcome generated by the immuno-PTT/PDT mediated by ICG-based nanomaterials in the primary/secondary tumors and tumor recurrence.

Formulation	Immuno Therapy Agent	PTT/ PDT Agent	Therapeutic Effect and Memory	Ref
**R837- and ICG-loaded PLGA NPs**	R837; Anti-CTLA-4 Ab (non-loaded)	ICG	R837- and ICG-loaded PLGA NPs + Laser + Anti-CTLA-4 Ab caused primary tumor eradication; R837- and ICG-loaded PLGA NPs + Laser + Anti-CTLA-4 Ab caused the greatest secondary tumor growth reduction; Metastases after R837- and ICG-loaded PLGA NPs + Anti-CTLA-4 Ab + Laser treatment decrease compared to control.	[38]
			Tumor-bearing mice previously treated with R837- and ICG-loaded PLGA NPs + anti-CTLA-4 Ab + Laser have reinoculated tumors with the slowest growth.	
**ICG- and RB-loaded DSPE-PEG-mal functionalized UCNPs**	Anti-CTLA-4 Ab (non-loaded)	ICG; RB	ICG- and RB-loaded DSPE-PEG-mal functionalized UCNPs + Laser with and without Anti-CTLA-4 Ab caused primary tumor eradication while the other treatment groups only caused tumor growth reduction; ICG- and RB-loaded DSPE-PEG-mal functionalized UCNPs + Laser + Anti-CTLA-4 Ab caused the strongest secondary tumor growth reduction; Metastases decrease after ICG- and RB-loaded DSPE-PEG-mal functionalized UCNPs + Laser + Anti-CTLA-4 Ab treatment.	[114]
			Tumor-bearing mice previously treated with ICG- and RB-loaded DSPE-PEG-mal functionalized UCNPs with Anti-CTLA-4 Ab + Laser have reinoculated tumors with the slowest growth.	
**Mg and ICG-loaded PES NPs**	-	ICG	Mg and ICG-loaded PES NPs + Laser caused tumor regression while the other treatment groups only caused tumor growth reduction; Mg and ICG-loaded PES NPs + Laser caused a great secondary tumor growth reduction compared to the other treatment groups; The number of metastatic nodules after Mg and ICG-loaded PES NPs + Laser treatment strongly decreases compared to control (3.39 vs. 41.53).	[32]
**ICG-loaded COF coated with ovalbumin**	Anti-PD-L1 Ab (non-loaded)	ICG; COF	ICG-loaded COF coated with ovalbumin + Laser, with and without Anti-PD-L1 Ab both caused primary tumor eradication; ICG-loaded COF coated with ovalbumin + Laser + Anti-PD-L1 Ab caused secondary tumor eradication while the other treatment groups only caused tumor growth reduction.	[73]
			Metastases after ICG-loaded COF coated with ovalbumin + Laser + Anti-PD-L1 Ab do not occur in mice after tumor reinoculation.	
**ICG-loaded liposome**	Anti-PD-1 Ab (non-loaded); Anti-TIM-3 Ab (non-loaded)	ICG	ICG-loaded liposome + Laser caused primary tumor eradication; ICG-loaded liposome + Laser + anti-PD-1 Ab + anti-TIM-3 Ab caused the strongest secondary tumor growth inhibition while the other treatment groups only caused tumor growth reduction.	[113]
**ICG-loaded PEG-Epacadostat conjugate NPs**	Epacadostat; Anti-PD-L1 Ab (non-loaded)	ICG	ICG-loaded PEG-Epacadostat conjugate NPs + Laser + Anti-PD-L1 Ab caused primary tumor eradication while the other treatment groups only caused tumor growth reduction; ICG-loaded PEG-Epacadostat conjugate NPs + Laser + Anti-PD-L1 Ab caused the strongest secondary tumor growth reduction.	[85]
**R837-loaded PEG-ICG Derivative ^(a)^-Cholic Acid based NPs ^(b)^**	R837; Anti-PD-1 Ab (non-loaded)	ICG derivative	R837-loaded PEG-ICG Derivative-Cholic Acid and PEG-Cysteine-Lysine-Cholic Acid based NPs + Laser + Anti-PD-1 Ab caused primary and secondary tumor eradication while the other treatment groups only caused tumor growth reduction.	[39]
**ICG-loaded lipid-PLGA NPs decorated with FimH**	FimH	ICG	ICG-loaded lipid-PLGA NPs decorated with FimH + Laser caused primary tumor eradication while the other treatment groups only caused tumor growth reduction.	[115]
			Metastases after ICG-loaded lipid-PLGA NPs decorated with FimH + Laser treatment do not occur in mice after tumor reinoculation.	
**CpG ODNs-loaded ICG functionalized MOF**	CpG ODNs	ICG	CpG-loaded ICG functionalized MOF + Laser caused primary tumor eradication while the other treatment groups only caused tumor growth reduction.	[120]
			Metastases after CpG-loaded ICG functionalized MOF + Laser treatment decrease in mice after tumor reinoculation.	
**ICG and poly I:C ^(c)^-loaded liposomes ^(d)^**	poly I:C	ICG	ICG and poly I:C-loaded liposomes + Laser. and ICG-loaded liposomes + Laser caused primary tumor regression while the other treatment groups do not reduce tumor growth.	[121]
			Metastases after ICG and poly I:C-loaded liposomes + Laser treatment strongly decrease compared to control in mice after tumor reinoculation.	
**ICG-loaded PLGA based NPs incorporated into decitabine, DSPE-PEG, and cell membranes based NPs**	Decitabine	ICG	ICG-loaded PLGA based NPs incorporated in decitabine, DSPE-PEG, and cell membranes based NPs + Laser caused primary tumor regression while the other treatment groups only reduce tumor growth; ICG-loaded PLGA based NPs incorporated in decitabine, DSPE-PEG, and cell membranes based NPs + Laser caused secondary tumor growth inhibition while the other treatment groups only caused tumor growth reduction.	[116]
**CAT, DTA-1 and ICG functionalized PDA NPs**	CAT	ICG; PDA	CAT, DTA-1, and ICG functionalized PDA NPs + Laser caused primary tumor regression while the other treatment groups only caused tumor growth reduction; CAT, DTA-1, and ICG functionalized PDA NPs + Laser caused the strongest secondary tumor growth reduction.	[117]
**ICG-loaded Mn@CaCO_3_ NPs functionalized with siPD-L1 and PAH**	siPD-L1	ICG	ICG-loaded siPD-L1 and PAH functionalized Mn@CaCO_3_ NPs + Laser caused primary tumor regression while the other treatment groups only caused tumor growth reduction.	[118]
**Anti-PD-L1 Ab and ICG-loaded PEG-PLGLAG-dEGCG NPs**	Anti-PD-L1 Ab	ICG	Anti-PD-L1 Ab and ICG-loaded PEG-PLGLAG-dEGCG NPs + Laser caused primary tumor growth inhibition while the other treatment groups only caused tumor growth reduction; Metastases after Anti-PD-L1 Ab and ICG-loaded PEG-PLGLAG-dEGCG NPs + Laser treatment do not occur (nodules: 0 vs. 16.17, treated vs. control).	[107]
			Tumor-bearing mice previously treated with Anti-PD-L1 Ab and ICG-loaded PEG-PLGLAG-dEGCG NPs have reinoculated tumors with the slowest growth.	
**ICG and R837-loaded HA functionalized ZIF-8 NPs**	R837	ICG	ICG and R837-loaded HA functionalized ZIF-8 NPs + Laser caused the strongest primary and secondary tumor growth reduction.	[108]
			Tumor-bearing mice previously treated with ICG and R837-loaded HA functionalized ZIF-8 NPs + Laser have reinoculated tumors with the slowest growth.	
**ICG and R837-loaded PEG-polyphenols functionalized Fe_3_O_4_ based NPs**	R837	ICG	ICG and R837-loaded PEG-polyphenols functionalized Fe_3_O_4_ based NPs + Laser caused the strongest primary tumor growth reduction.	[69]
			Tibia and lung metastases after ICG and R837-loaded PEG-polyphenols functionalized Fe_3_O_4_ based NPs + Laser treatment strongly decrease in mice after tumor reinoculation compared to the other treatment groups.	
**ICG and PM-loaded albumin MnO_2_ NPs**	PM; MnO_2_	ICG	ICG and PM-loaded albumin MnO_2_ NPs caused the strongest primary and secondary tumor growth reduction.	[110]
**FAL-PEG-TA and PEI-ICG functionalized AuNS and Hb-loaded FAL liposomes**	Hb	ICG; AuNS	FAL-PEG-TA and PEI-ICG functionalized AuNS and Hb-loaded FAL liposomes + Laser caused the strongest primary tumor growth reduction.	[76]
**ICG-loaded PLGA NPs incorporated in EPV**	EPV	ICG	ICG-loaded PLGA NPs incorporated in EPV + Laser caused the strongest primary tumor growth reduction.	[119]
**ICG, R837, and CTL-Ap-loaded dextran NPs**	R837; CTL-ap	ICG	ICG, R837, and CTL-Ap-loaded dextran NPs + Laser caused the strongest primary tumor growth reduction.	[109]
**ICG and R837-IONs-loaded DSPE-PEG NPs**	R837; IONs	ICG	The number of metastatic nodules after ICG and R837-IONs-loaded DSPE-PEG NPs + Laser treatment severely decreases compared to control (3.46 vs. 22.30).	[99]

^(a)^ Functionalized with a carboxylic acid group; ^(b)^ Based in PEG-Cysteine-Lysine-Cholic Acid; ^(c)^ Polyinosinic:polycytidylic Acid; ^(d)^ Formulated with DPPC, 1-Palmitoyl-2-Hydroxy-*sn*-Glycero-3-Phosphocholine, and DSPE-PEG.

**Table 3:** Outcome generated by the immuno-PTT/PDT mediated by prototypic HC-based nanomaterials in the levels of mDCs and T cells.

Formulation	Immuno Therapy Agent	PTT/ PDT Agent	Changes in the Levels of mDCs and T Cells	Ref
**Met- ^(a)^ and IR775-loaded liposomes ^(b)^**	Met	IR775	Met- and IR775-loaded liposomes + Laser induced 2.5, 2.21, 2.14, 3.96, and 4.49 times higher CTLs levels than Met- and IR775-loaded liposomes, Met-loaded liposomes, IR775-loaded liposomes + Laser, control + Laser, and the control, respectively (in the primary tumor); Met- and IR775-loaded liposomes + Laser induced 2.27, 4.17, 2.21, 4.69, and 4.69 times higher CTLs levels than Met and IR775-loaded liposomes, Met-loaded liposomes, IR775-loaded liposomes + Laser, control + Laser, and the control, respectively (in the secondary tumor).	[72]
**NLG919- and IR780-loaded PEG-PCL micelles**	NLG919	IR780	NLG919- and IR780-loaded PEG-PCL micelles + Laser induced 6.79, 33.12, 11.04, and 43.72 times higher CTLs/T_reg_ ratio than NLG919 and IR780-loaded PEG-PCL micelles, IR780-loaded PEG-PCL micelles + Laser, NLG919-loaded PEG-PCL micelles, and the control, respectively (in spleen).	[106]
**IR780 and Imatinib-loaded PEGylated GITR-functionalized PLGA based NPs**	Imatinib	IR780	IR780- and Imatinib-loaded PEGylated GITR-functionalized PLGA based NPs + Laser induced about two times higher mDC levels than IR780 + Laser, Imatinib, control + Laser, and the control, respectively (in the primary tumor).	[71]
			IR780- and Imatinib-loaded PEGylated GITR-functionalized PLGA-based NPs + Laser induced 2.54, 2.34, 3.25, and 3.38 times lower T_reg_ levels than IR780 + Laser, Imatinib, control + Laser, and the control, respectively (in the primary tumor).	
**IR780- and Met-loaded CeO_2_-capped MSNs ^(c)^**	Met; CeO_2_	IR780	IR780- and Met-loaded CeO_2_-capped MSNs + Laser induced 1.06, 1.39, 2.10, and 1.58 times higher CTLs levels than IR780 and Met-loaded MSNs + Laser, IR780-loaded CeO_2_-capped MSNs + Laser, IR780-loaded MSNs + Laser, and the control, respectively (in the primary tumor).	[88]
**BMS- ^(d)^-loaded IR780-PEGylated lipidic ^(e)^ NPs**	BMS	IR780	BMS-loaded IR780-PEGylated lipidic NPs + Laser induced about 1.30 times higher mDC levels than IR780-PEGylated lipidic NPs + Laser, BMS-loaded lipidic NPs + Laser, and BMS + Laser (in lymph nodes); BMS-loaded IR780-PEGylated lipidic NPs + Laser induced 1.9 times higher mDC levels than the control (in lymph nodes).	[33]
			BMS-loaded IR780-PEGylated lipidic NPs + Laser induced 1.69, 2, 2.34, and 3.58 times higher CTLs levels than IR780-PEGylated lipidic NPs + Laser, BMS-loaded lipidic NPs + Laser, BMS + Laser, and the control, respectively (in the primary tumor).	
**IR780- and SB-505124-loaded liposomes ^(f)^**	SB-505124	IR780	IR780- and SB-loaded liposomes + Laser induced 1.61, 1.16, 1.76, and 2.24 times higher CTLs levels than IR780-loaded liposomes + Laser, IR780 and SB-loaded liposomes, SB, and the control, respectively (in the primary tumor); IR780- and SB-loaded liposomes + Laser induced 1.91, 1.22, 1.68, and 2.28 times lower T_reg_ levels than IR780-loaded liposomes + Laser, IR780 and SB-loaded liposomes, SB, and the control, respectively (in the primary tumor).	[74]
**IR780-loaded PEG-PCL NPs**	-	IR780	IR780-loaded PEG-PCL NPs + Laser induced 1.22 and 1.44 times higher mDC levels than IR780 + Laser, and the control, respectively (in the primary tumor).	[122]
			IR780-loaded PEG-PCL NPs + Laser induced 1.44 and 2 times higher CTLs levels than IR780 + Laser, and the control, respectively (in the primary tumor).	
**IR797-loaded DSPE-PEG NPs coated with mDCs membranes ^(g)^**	mDCs membranes	IR797	IR797-loaded DSPE-PEG NPs coated with mDCs membranes + Laser induced 1.21 and 2.05 times higher mDC levels than IR797-loaded DSPE-PEG NPs coated with mDCs membranes and IR797-loaded DSPE-PEG NPs + Laser, respectively (in lymph nodes); IR797-loaded DSPE-PEG NPs coated with mDCs membranes + Laser induced about 2.60 times higher mDC levels than IR797-loaded DSPE-PEG NPs and the control (in lymph nodes).	[65]
			IR797-loaded DSPE-PEG NPs coated with mDCs membranes + Laser induced 1.26 times higher CTLs levels than IR797-loaded DSPE-PEG NPs coated with mDCs membranes (in the primary tumor); IR797-loaded DSPE-PEG NPs coated with mDCs membranes + Laser induced about 4.8 times higher CTLs levels than IR797-loaded DSPE-PEG NPs + Laser, IR797-loaded DSPE-PEG NPs, and the control (in the primary tumor); IR797-loaded DSPE-PEG NPs coated with mDCs membranes + Laser induced 1.31, 2.48, 2.77, and 3.79 times higher CTLs levels than IR797-loaded DSPE-PEG NPs coated with mDCs membranes, IR797-loaded DSPE-PEG NPs + Laser, IR797-loaded DSPE-PEG NPs, and the control, respectively (in the secondary tumor).	
**IR820-loaded HA functionalized MOF NPs ^(h)^, and R837 and 1MT-loaded mannan functionalized MOF NPs ^(h)^**	R837; 1MT	IR820	IR820-loaded HA functionalized MOF NPs + R837 and 1MT-loaded mannan functionalized MOF NPs + Laser induced 2.32, 1.96, and 10.22 times higher CTLs/T_reg_ ratios than IR820-loaded HA functionalized MOF NPs + Laser, R837 and 1MT-loaded mannan functionalized MOF NPs, and the control, respectively (in the primary tumor); IR820-loaded HA functionalized MOF NPs + R837 and 1MT-loaded mannan functionalized MOF NPs + Laser induced 3.84, 3.80, and 6.82 times higher CTLs/T_reg_ ratios than IR820-loaded HA functionalized MOF NPs + Laser, R837 and 1MT-loaded mannan functionalized MOF NPs, and the control, respectively (in splenic lymphocytes); IR820-loaded HA functionalized MOF NPs + R837 and 1MT-loaded mannan functionalized MOF NPs + Laser induced 2.71, 2, and 10 times higher CTLs/T_reg_ ratios than IR820-loaded HA functionalized MOF NPs + Laser, R837 and 1MT-loaded mannan functionalized MOF NPs, and the control, respectively (in the secondary tumor).	[37]
			IR820-loaded HA functionalized MOF NPs + R837 and 1MT-loaded mannan functionalized MOF NPs + Laser generate 2.29, 1.75, and 4 times higher memory T cells levels than HA-functionalized MOF NPs + Laser, R837 and 1MT-loaded mannan-functionalized MOF NPs, and the control, respectively (in splenic lymphocytes).	
**1MT-IR820 NPs**	1MT; Anti-PD-L1 Ab (non-loaded)	IR820	1MT-IR820 NPs + Laser + Anti-PD-L1 Ab induced 1.18, 1.40, 1.51, 1.92, and 2.38 times higher mDC levels than 1MT-IR820 NPs + Laser, IR820 + Laser, Anti-PD-L1 Ab, 1MT, and the control, respectively (in lymph nodes).	[75]
			1MT-IR820 NPs + Laser + Anti-PD-L1 Ab induced 1.54, 1.97, 3.55, 4.73, and 6.45 times higher CTLs/T_reg_ ratio than 1MT-IR820 NPs + Laser, IR820 + Laser, Anti-PD-L1 Ab, 1MT, and the control, respectively (in the primary tumor); 1MT-IR820 NPs + Laser + Anti-PD-L1 Ab induced 1.35, 1.64, 1.97, 2.42, and 3.38 times higher CTLs levels than 1MT-IR820 NPs + Laser, IR820 + Laser, Anti-PD-L1 Ab, 1MT, and the control, respectively (in the secondary tumor).	
			1MT-IR820 NPs + Laser + Anti-PD-L1 Ab generated 1.19, 1.36, 1.73, 1.93, and 2.24 times higher T_EM_ cells levels than 1MT-IR820 NPs + Laser, IR820 + Laser, Anti-PD-L1 Ab, 1MT, and the control, respectively (in spleens).	

^(a)^ Metformin; ^(b)^ Formulated with Hydrogenated Soybean Phosphatidylcholine (HSPC), cholesterol, and DSPE-PEG; ^(c)^ Mesoporous Silica NPs; ^(d)^ PD-1/PD-L1 inhibitor BMS202; ^(e)^ Formulated with DPPC and cholesterol; ^(f)^ Formulated with DSPE-PEG, DPPC and cholesterol; ^(g)^ Obtained by exposing immature DCs to TAAs and Poly I:C; ^(h)^ Based on ZIF-8.

**Table 4:** Outcome generated by the immuno-PTT/PDT mediated by prototypic HC-based nanomaterials in the primary/secondary tumors and tumor recurrence.

Formulation	Immuno Therapy Agent	PTT/ PDT Agent	Therapeutic Effect and Memory	Ref
**Met- and IR775-loaded liposomes**	Met	IR775	Met- and IR775-loaded liposomes + Laser caused the strongest primary and secondary tumor growth reduction.	[72]
**NLG919- and IR780-loaded PEG-PCL micelles**	NLG919	IR780	NLG919- and IR780-loaded PEG-PCL micelles + Laser caused primary tumor eradication while the other treatment groups only caused tumor growth reduction; NLG919- and IR780-loaded PEG-PCL micelles + Laser caused the strongest secondary tumor growth reduction; Metastases after NLG919- and IR780-loaded PEG-PCL micelles + Laser treatment decreased compared to the control.	[106]
**IR780- and Imatinib-loaded PEGylated GITR-functionalized PLGA-based NPs**	Imatinib	IR780	IR780 and Imatinib-loaded PEGylated GITR-functionalized PLGA-based NPs caused primary tumor eradication while the other treatment groups only caused tumor growth reduction.	[71]
**IR780- and Met-loaded CeO_2_-capped MSNs**	Met; CeO_2_	IR780	IR780- and Met-loaded CeO_2_-capped MSNs + Laser caused primary tumor regression while the other treatment groups only caused tumor growth reduction; The number of metastatic nodules after IR780 and Met-loaded CeO_2_-capped MSNs + Laser treatment severely decreased compared to control (about 5 vs. 58).	[88]
**BMS-loaded IR780-PEGylated lipidic NPs**	BMS	IR780	BMS-loaded IR780-PEG lipidic NPs + Laser caused primary tumor regression while other treatment groups only caused tumor growth reduction; The number of metastatic nodules after BMS-loaded IR780-PEG lipidic NPs + Laser treatment severely decreased compared to the control (about 4.60 vs. 52).	[33]
**IR780- and SB-505124-loaded liposomes**	SB-505124; Anti-PD-1 Ab (non-loaded)	IR780	IR780- and SB-loaded liposomes + Laser + Anti-PD-1 caused primary tumor growth inhibition while the other treatment groups only caused tumor growth reduction; IR780- and SB-loaded liposomes + Laser + Anti-PD-1 caused the strongest secondary tumor growth reduction; The number of metastatic nodules after IR780 and SB-loaded liposomes + Laser + Anti-PD-1 treatment severely decreased compared to the control (about 2.80 vs. 38).	[74]
**IR780-loaded PEG-PCL NPs**	-	IR780	IR780-loaded PEG-PCL NPs + Laser induced primary tumor growth reduction; Metastases after IR780-loaded PEG-PCL NPs + Laser treatment decreased compared to the control.	[122]
**IR797-loaded DSPE-PEG NPs coated with mDC membranes**	mDCs membranes	IR797	IR797-loaded DSPE-PEG NPs coated with mDCs membranes + Laser caused primary tumor eradication while other treatment groups only caused tumor growth reduction; IR797-loaded DSPE-PEG NPs coated with mDCs membranes + Laser caused the strongest secondary tumor growth reduction.	[65]
**IR820-loaded HA-functionalized MOF NPs, and R837- and 1MT-loaded mannan-functionalized MOF NPs**	R837; 1MT	IR820	IR820-loaded HA-functionalized MOF NPs + R837 and 1MT-loaded mannan-functionalized MOF NPs + Laser caused primary tumor regression while the other treatment groups only caused tumor growth reduction; IR820-loaded HA-functionalized MOF NPs + R837 and 1MT-loaded mannan-functionalized MOF NPs + Laser caused the strongest secondary tumor growth reduction.	[37]
			Metastases after IR820-loaded HA-functionalized MOF NPs + R837 and 1MT-loaded mannan-functionalized MOF NPs + Laser treatment strongly decreased in mice after tumor reinoculation compared to the control.	
**AG ^(a)^-IR820 conjugate, Quercetin-loaded liposomes ^(b)^, and LPS ^(c)^**	LPS (non-loaded)	IR820	AG-IR820 + Quercetin-loaded liposomes + Laser + LPS caused primary tumor regression while the other treatment groups only caused tumor growth reduction.	[124]
**1MT-IR820 NPs**	1MT; Anti-PD-L1 Ab (non-loaded)	IR820	1MT-IR820 NPs + Laser + Anti-PD-L1 Ab caused the strongest primary and secondary tumor growth reduction.	[75]
			Metastases after 1MT-IR820 NPs + Laser + Anti-PD-L1 Ab treatment strongly decreased in mice after tumor reinoculation compared to the control.	
**IR820-loaded DSPE-PEG-TPP ^(d)^ and DSPE-PEG-CpG ODNs-functionalized GO ^(e)^**	DSPE-PEG-CpG	GO; IR820	IR820-loaded DSPE-PEG-TPP and DSPE-PEG-CpG ODNs-functionalized GO + Laser induced the strongest primary tumor growth reduction.	[95]

^(a)^ Amino-glucose; ^(b)^ Formulated with HSPC, Cholesterol and PEGylated phosphatidylethanolamine; ^(c)^ Lipopolysaccharide; ^(d)^ Triphenylphosphonium; ^(e)^ Graphene Oxide.

## Data Availability

Data sharing not applicable.
